# From “warning” to “guidance”: design of a closure control zone for eight-lane freeways and evaluation of its regulating effects on driving behavior

**DOI:** 10.3389/fpubh.2026.1803755

**Published:** 2026-04-16

**Authors:** Xiaojun Zhao, Runzhao Bei, Jing Zhang, Yuan Ling, Xiangjun Yang

**Affiliations:** 1 Xinjiang Jiaotou Construction Management Co., Ltd., Urumchi, China; 2 Intelligent Transportation Systems Research Center, Wuhan University of Technology, Wuhan, China; 3 Xinjiang Transportation Research Institute Co., Ltd., Urumchi, China; 4 Xinjiang Transportation Dukou Expressway Investment and Development Co., Ltd., Urumchi, China

**Keywords:** driving behavior, driving simulation, eight-lane freeway, mainline closure, road traffic safety, warning sign

## Abstract

**Introduction:**

Emergency closures of eight-lane freeway mainlines require vehicles to execute multiple successive lane changes to detour, a maneuver that is more complex and risky than on narrower freeways. Current standards lack tailored control zone designs and empirical evidence for such scenarios.

**Methods:**

To address the lack of standards for eight-lane freeway full closures, a control zone was designed based on the principle of “active guidance, graded transition, and terminal enforcement.” Recognizing the complexity of multiple mandatory lane changes, two warning signs were developed: a basic version and an enhanced version adding the advisory “Merge Right for Detour.” A 2 × 2 factorial driving simulator experiment (sign type × weather condition) involving 30 licensed drivers was conducted to evaluate lateral and longitudinal driving behavior.

**Results:**

The enhanced “Warning & Advice” sign consistently improved driving performance. It prompted earlier lane-change initiation (by 966 m in clear weather and 610 m in dusty weather) and reduced the aggressiveness of three successive lane changes (by 35.8, 23.8, and 23.5% in clear weather; the first two by 49.3 and 31.3% in dusty weather). Longitudinally, it decreased maximum deceleration aggressiveness by 21.7% (clear) and 25.1% (dust) and improved deceleration smoothness by 16.0 and 25.4%, respectively. Dusty weather amplified these benefits, indicating a synergy between environmental caution and explicit guidance.

**Discussion:**

Incorporating actionable advice into warning signs optimizes driver decision-making and shifts the control strategy from passive “warning” to active “guidance.” The findings provide an empirical basis for the design of closure control zones and demonstrate that well-designed information can compensate for environmental uncertainty, thereby enhancing safety in complex multi-lane closures.

## 1 Introduction

The closure of a freeway mainline due to an emergency, such as extreme weather or a major traffic incident, is a high-risk scenario in road operation and management. It necessitates the safe and efficient diversion of traffic via an upstream exit. Current primary standards and guidelines, including China’s Road Traffic Signs and Markings (GB-5768.3-2025) and Specifications for Safety Operation of Highway Maintenance (JTG-H30-2015), as well as the US Manual on Uniform Traffic Control Devices (MUTCD), primarily provide control strategies for work zones or traffic incident management areas where only partial lanes are closed. For full mainline closures, the common approach involves long-term measures like adding orange arrows to advance exit guide signs or overhead directional signs. In practice, traffic management agencies often resort to adapting temporary work zone setups, which can lead to issues such as arbitrarily placed channelizing devices and unclear sign information, thereby increasing uncertainty in control effectiveness. This gap between standards and practical needs is critically amplified on eight-lane freeways. Here, vehicles may need to change lanes up to four times to exit, making the maneuver significantly more complex and the risk of traffic conflicts much higher than on traditional four- or six-lane freeways (1). Consequently, systematic research into the design of a dedicated closure control zone for eight-lane freeways is urgently needed to enhance emergency management capabilities on high-grade roads.

Existing theories and methods related to closure control primarily stem from two fields: standards and practices for maintenance work zones, and research on traffic control devices and driving behavior. Regarding work zone control, both Chinese and US standards (JTG-H30-2015 and MUTCD) establish a functional zoning framework centered on advance warning, transition, and buffer areas, with detailed specifications for key parameters like speed reduction gradients and spacing of channelizing devices, providing a foundational safety guarantee. However, the design logic of these standards is based on scenarios involving partial lane closures or planned long-term mainline closures on four- or six-lane freeways. For instance, while JTG-H30-2015 guides drivers to gradually detect the zone, decelerate, change lanes, and buffer through its designated areas, it does not fully account for the unique requirement of multiple mandatory lane changes during an unexpected full closure on an eight-lane freeway. Furthermore, the standard warning signs used in work zones are ill-suited for conveying the nature and urgency of emergencies (e.g., “closed due to high winds”) and fail to provide clear operational guidance, potentially leading to inadequate risk perception and driver hesitation.

Recent research on driving behavior and traffic signs offers valuable theoretical support but also reveals limitations in addressing complex closure scenarios. Regarding information presentation, studies indicate that graphical or animated signs are more effective than traditional text-based signs in capturing attention and guiding behavior (2, 3). More instructively, messages containing specific action advice (e.g., “Prepare to Stop”) can prompt more timely speed adjustment and lane-changing behavior compared to vague warnings (4). In particular, innovative traffic control devices have shown promise in enhancing driver performance. Shi et al. demonstrated that self-luminous road markings can significantly improve driver behavior at unsignalized intersections, reducing vehicle speeds by approximately 20% and enhancing hazard perception (5, 6).

Concerning environmental influences, research confirms that adverse weather (e.g., fog, dust) not only alters drivers’ speed choices (7) but also increases their reliance on clear, anticipatory guidance information (8). Methodologically, recent advances in driving behavior quantification provide robust foundations for empirical investigation. Luo et al. proposed a multidimensional risk measurement framework for heavy-duty trucks using trajectory data, integrating lateral position, speed, and acceleration to construct quantitative indicators of driving stability and car-following risk (9). This work demonstrates the power of fusing multiple behavioral dimensions to systematically characterize driving dynamics—an approach that informs the indicator system developed in our study. Furthermore, in the context of lane-changing behavior, Luo et al. developed a weather-aware risk resilience assessment model that incorporates both Risk Exposure Level (REL) and Risk Severity Level (RSL), showing that weather conditions significantly modulate lane-change risk and require adaptive modeling (10). Their finding directly validates our decision to include weather as a core experimental variable.

Nonetheless, most of these studies have contextual limitations: they focus on urban roads (11), intersections (12), or general lane reductions (13), lacking systematic exploration of the extreme scenario of a full closure on an eight-lane freeway. Others concentrate on the location and format of information (14, 15) without deeply dissecting the differential impact of information content hierarchy within complex sequences of driving tasks. While the aforementioned studies have laid important groundwork—demonstrating the effectiveness of enhanced information facilities (5, 6) and establishing rigorous methods for quantifying driving risk under varying conditions (9, 10)—the specific context of emergency full closures on eight-lane freeways remains underexplored. In this scenario, drivers face the compounded challenge of multiple successive mandatory lane changes under time pressure and, potentially, adverse weather. The question of how to design warning signs that optimally guide drivers through this complex task sequence, and whether such designs interact with environmental conditions, has yet to be systematically addressed.

In summary, two critical gaps exist in current research and practice: First, regarding the subject, there is a lack of a systematic control zone design scheme, from information guidance to physical channelization, specifically tailored for emergency closure scenarios on eight-lane freeways. Second, methodologically, there is insufficient experimental validation on how to optimize the content of warning signs to manage the interaction between the demanding driving task of “multiple lane changes” and the environmental pressure of “adverse weather.” Therefore, this study aims to address these gaps by focusing on the following core scientific question: How can a closure control zone scheme be designed for eight-lane freeways, and how can the precise design of warning sign content effectively mitigate drivers’ behavioral risks during sudden closures under adverse weather conditions?

To this end, this study undertakes the following innovative work: (1) Proposes a systematic design concept for a “freeway closure control zone,” clarifying the design principles of “information-driven, active guidance; task decomposition, graded transition; terminal enforcement, ensured diversion,” and determining key design parameters; (2) Designs two types of warning signs (basic warning vs. warning with advisory action) based on drivers’ information needs in emergencies and empirically tests their effectiveness under different weather conditions through a driving simulator experiment; (3) Constructs five quantitative indicators covering lane-changing aggressiveness, deceleration aggressiveness, and process smoothness from both lateral and longitudinal behavioral safety dimensions to precisely evaluate the impact of weather conditions and warning sign content on driving safety.

The structure of this paper is as follows: Section 1 details the design of the closure control zone and the driving simulation experimental methodology. Section 2 presents the driving behavior safety results under different experimental scenarios. Section 3 provides an in-depth discussion of the results. Section 4 summarizes the main conclusions and outlines directions for future research.

## 2 Methodology

### 2.1 Design of the closure control zone for eight-lane freeways

To address the complex requirement for multiple lane changes during a full freeway closure, this study proposes a design principle centered on “active guidance, graded transition, and terminal enforcement,” building upon the traditional work zone control framework. Conventional work zone management follows a reactive sequence of “detection → mandatory deceleration → mandatory lane change,” often resulting in drivers responding passively to channelizing devices only upon reaching the end of the advance warning area. By optimizing the information structure at the outset of the warning area, this study shifts the control logic to a proactive guidance model: “cognition → planning → guided lane change → mandatory deceleration → enforced diversion.” Specifically, at the upstream end of the warning area, a warning sign containing explicit action advice prompts drivers to recognize the hazard early and begin planning and executing lane changes. This effectively distributes and front-loads the task of completing four lane changes. The subsequent transition and buffer areas then assume and complete any remaining lane-change maneuvers. These areas use physical channelization to compel lane changes from non-compliant vehicles, ensuring all traffic is ultimately diverted at the designated exit point.

**Figure 1:**
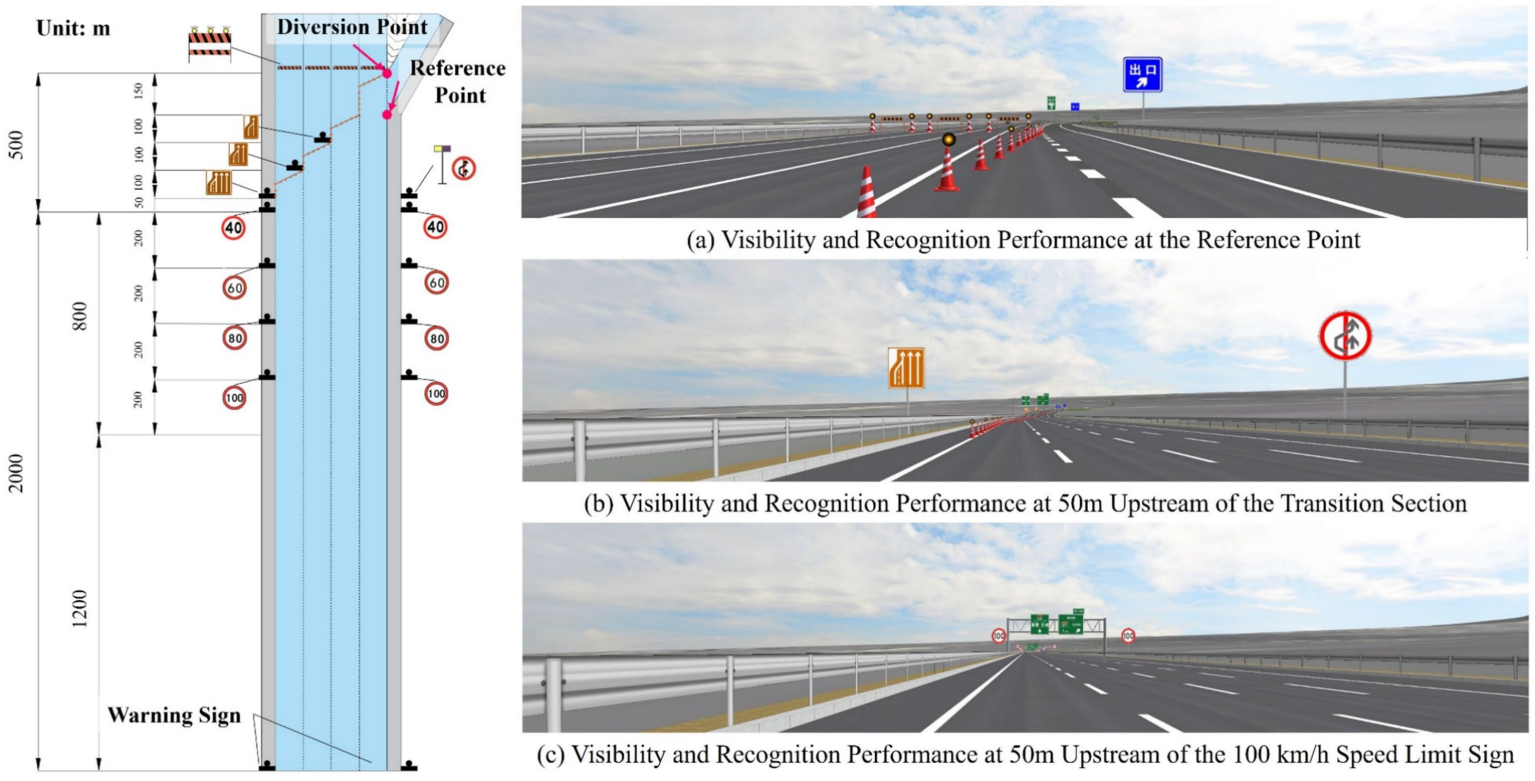
Layout and parameters of sections and related facilities within the closure control zone on an eight-lane freeway.

Guided by this principle and with reference to parameters from established standards, a closure control zone suitable for an eight-lane freeway (with a speed limit of 120 km/h) was designed; the overall layout is shown in Figure 1. The control zone is divided, from upstream to downstream, into a Warning Area, a Transition Area, and a Buffer Area.

1. Warning Area: Extending 2,000 meters from the control zone origin to the start of the transition area, this section informs drivers of the closure and guides pre-emptive behavioral adjustments. A warning sign is placed at its beginning. Commencing 1,000 meters upstream of the transition area, a series of graded speed limit signs (100, 80, 60, and 40 km/h) are installed at 200-meter intervals to guide vehicles through a gradual and smooth deceleration.
2. Transition and Buffer Areas: These sections are critical for enforcing mandatory lane changes and employ traffic cones for physical channelization. Cones are spaced 4 meters apart, with a yellow warning light installed at every third cone. The cones are placed 0.4 meters from the right-hand lane edge. The Transition Area extends upstream from the base point and consists of three structural units. Each unit contains a 50-meter lane-change section followed by a 50-meter adjustment section to facilitate a smooth lateral shift of traffic flow. The Buffer Area extends upstream from the diversion point. Its length equals the distance from the diversion point to the base point, and it includes a final 50-meter lane-change section to provide a last opportunity for vehicles to merge. A “Lane Ends” sign is placed on the left roadside 50 meters upstream of the Transition Area, accompanied by a yellow/blue flashing light and a “No Passing” sign on the right. Additional “Lane Ends” signs are positioned at the end of each lane-change section, and an “Exit Guide” sign is placed at the terminus of the Transition Area.
3. Diversion Point: A row of barriers is installed as the terminal point of the Buffer Area, physically preventing vehicles from continuing straight ahead.

All specified parameters comply with human factors principles and the requirements of standards GB 5768.3–2025 and JTG H30-2015. As the core focus of this study is warning sign optimization, the derivation process for these geometric and spacing parameters is not elaborated here.

**Figure 2:**
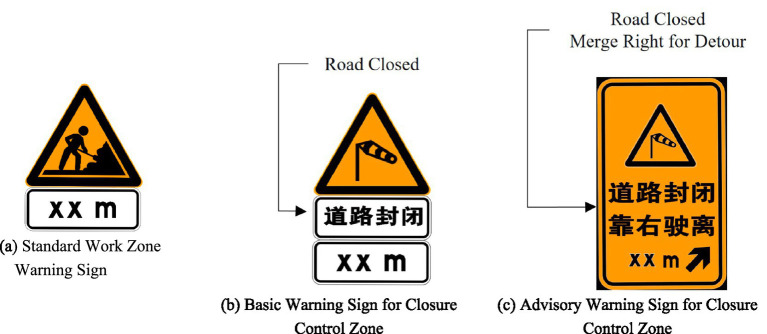
Three types of warning signs. **(a)** Standard work zone warning sign with a symbol and distance information. **(b)** Basic warning sign for the closure control zone, indicating “High Wind Ahead” and “Mainline Closed 2000m Ahead”. **(c)** Enhanced advisory warning sign, which adds the instruction “Keep Right to Exit” with a rightward arrow below the basic warning information.

To design effective warning signs for emergency closure control, this study first analyzed the information composition of standard work zone warning signs. As shown in Figure 2a, the typical sign at a work zone origin combines a work area symbol with distance information, clearly conveying three core messages: “work ahead,” “active zone control,” and “distance to control point.” Building on this framework, and considering the absence of dedicated signs for emergency scenarios, this study proposes that a warning sign at the closure control zone origin should contain at least three basic information elements: “incident type,” “closure control,” and “distance to control point.” This ensures a minimum level of warning effectiveness equivalent to standard work zone signs. Furthermore, recognizing the complex need for multiple lane changes on eight-lane freeways, an operational advisory—the text “Keep Right to Exit” accompanied by an arrow indicator—was added to this basic information to create an enhanced “Warning + Advice” sign. Accordingly, using a high-wind closure as an example, two warning sign variants were designed: one providing only the basic warning information, and the other incorporating the additional advisory (Figures 2b,c).

### 2.2 Experimental apparatus and participants

#### 2.2.1 Participants

A total of 30 licensed drivers successfully completed the experiment. The sample size was determined based on two considerations. First, the minimum required sample size was calculated using the formula for estimating population means: $N=Z^{2}\sigma^{2}/E^{2}$ , where *Z* = 1.645 (corresponding to a 90% confidence level), σ =0.40 (estimated standard deviation of key driving behavior indicators), and *E* = 0.10 (acceptable margin of error). This yielded a minimum requirement of approximately 17 participants (16). Second, a review of comparable driving simulator studies indicated that sample sizes typically do not exceed 30 participants (17, 18). Considering both the statistical requirement and prevailing practices in the field, 30 participants were recruited. The final cohort of 30 participants reflected the gender and age distribution of the general Chinese driver population (19, 20); their basic demographics are presented in Table 1. All participants had normal or corrected-to-normal vision, normal hearing, and possessed over 10,000 kilometers of practical freeway driving experience.

**Table 1:** Basic information of participants.

Driver characteristic	Number (N)	Percentage (%)
Male	20	66.67
Female	10	33.33
22–26 years old	5	16.67
26–50 years old	21	70
51–60 years old	4	13.33

Participant recruitment was conducted from July 1 to October 31, 2025. This study was conducted in accordance with the World Medical Association’s Declaration of Helsinki. The studies involving human participants were reviewed and approved by the Institutional Review Board of Wuhan University of Technology. All participants provided written informed consent prior to the experiment, which included consent for the publication of anonymized results. No identifying information of the participants is included in this manuscript.

#### 2.2.2 Experimental apparatus

**Figure 3:**
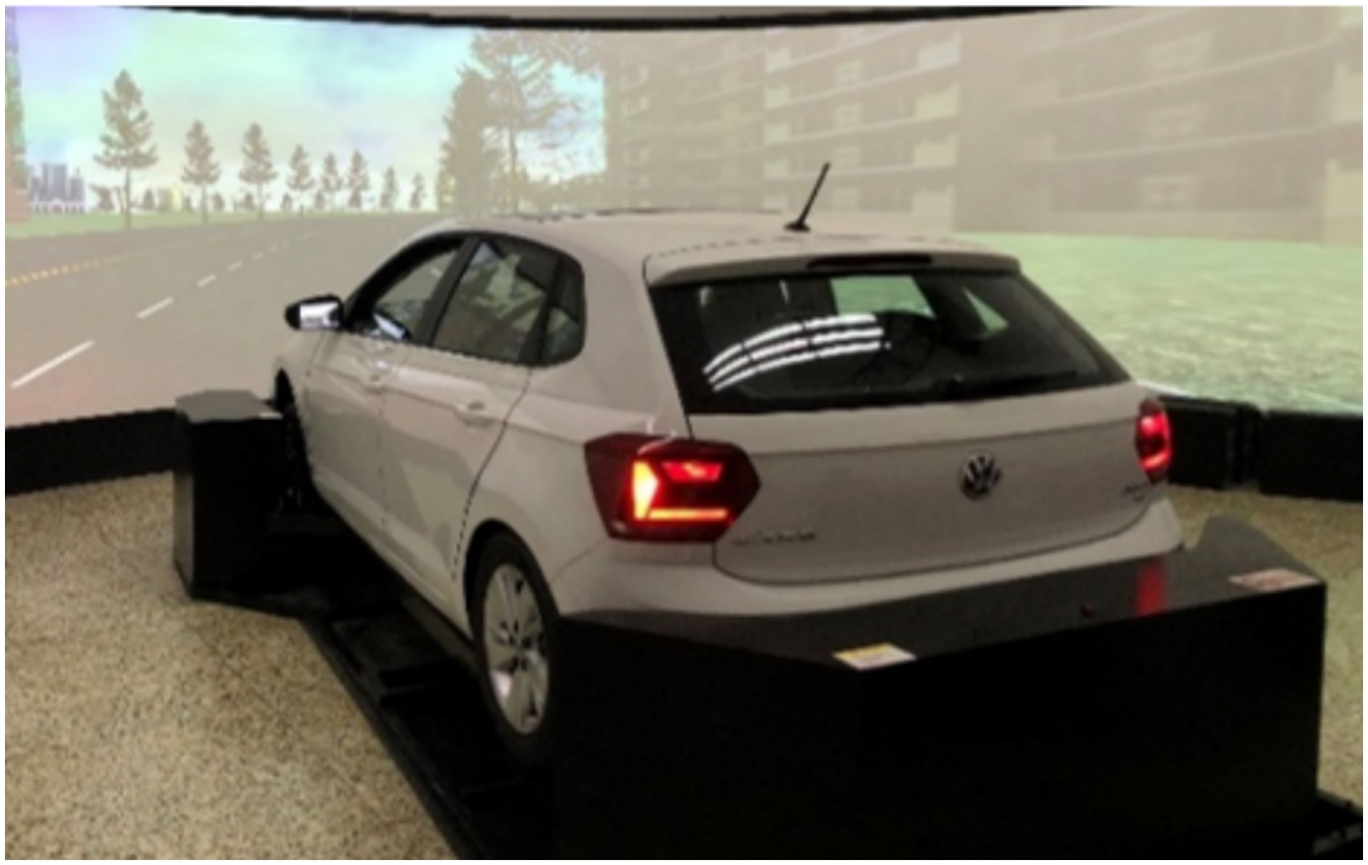
High-fidelity 3-DOF driving simulator platform.

The experiment was conducted using a high-fidelity driving simulator at Wuhan University of Technology, the overall structure of which is shown in Figure 3. The platform consists of three main subsystems: a driver cockpit, a three-degree-of-freedom (3-DOF) motion platform, and a visual projection system. The cockpit was modified from a real vehicle, providing a high degree of realism. It was instrumented with additional sensors integrated with the original Controller Area Network (CAN) bus to enable real-time collection of driving operation signals. The 3-DOF motion platform simulates longitudinal, lateral, and vertical translational movements, delivering a highly realistic vehicle control experience. The visual system comprises a 180-degree curved screen, five projectors, virtual side mirrors, and an image blending/correction unit, collectively creating an immersive simulation environment. The three-dimensional driving scenarios were developed and all driving behavior data were recorded using UC-win/Road software (version 16.0.1, represented by Forum8 Shanghai Co., Ltd.). Relevant data were logged at a frequency of 10 Hz. The validity and reliability of this type of simulation platform for driving behavior research have been established in previous studies (21–23).

It should be noted that while the study site is located in a high-wind area, the “high wind” condition serves as the contextual background for the emergency closure scenario rather than an experimentally simulated physical disturbance. The driving simulator experiment focused on driver behavioral responses to warning sign information and visibility conditions, with vehicle dynamics modeled under normal operational conditions.

### 2.3 Driving simulation experiment

#### 2.3.1 Driving simulation scenarios

**Figure 4:**
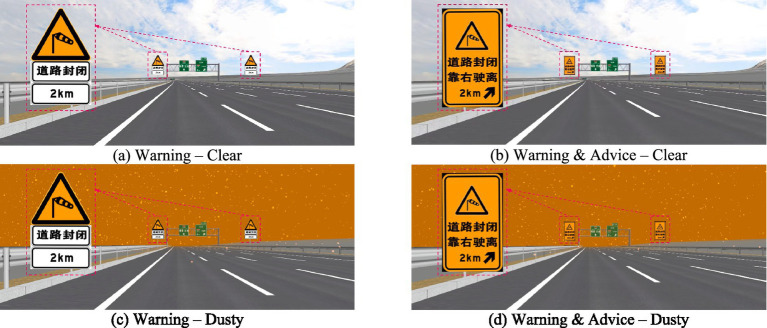
Warning sign types and weather conditions in the driving simulation experiment. From left to right, top to bottom: **(a)** Clear weather with the basic warning sign, **(b)** clear weather with the enhanced advisory sign, **(c)** dusty weather (low visibility) with the basic warning sign, **(d)** dusty weather with the enhanced advisory sign.

The driving simulation scenario was constructed based on a 29-km road model of an eight-lane freeway section, with each lane measuring 3.75 meters in width, recreated at 1:1 scale from design blueprints. The modeled segment includes 5 km upstream of the exit and 24 km downstream. Participants began each trial 5 km upstream of the exit, which is 2.5 km upstream of the closure control zone origin, and drove until reaching the exit point where the trial terminated. This design served two purposes: (1) to provide an immersive driving experience before encountering the warning sign, and (2) to avoid excessively long single trials that could induce fatigue or simulator sickness. The analysis focused solely on driver behavior within the 3-km closure control zone upstream of the exit. A 2 (Sign Type: Warning vs. Warning & Advice) × 2 (Weather Condition: Clear vs. Dust) within-subjects factorial design was employed. This resulted in four distinct driving scenarios to systematically evaluate the impact of warning sign content and weather on driving safety. Schematic representations of all experimental scenarios are provided in Figure 4. For the dust condition, visibility was set to 200 meters.

For the dust condition, visibility was set to 200 meters. This was achieved by adjusting the visual rendering parameters in UC-win/Road to simulate the optical effects of dusty weather, including reduced contrast, color desaturation, and atmospheric scattering. The realism of the simulated weather conditions was ensured through two approaches. First, the visual parameters were calibrated based on published standards for low-visibility driving simulation (24). Second, during the adaptation practice session, participants were exposed to the weather conditions and asked to verbally confirm that the visual appearance was consistent with their real-world driving experience in dusty environments. All participants reported that the simulation was realistic and acceptable. This approach aligns with established practices in driving simulator research, where subjective verification by participants is commonly used to validate environmental realism (25).

#### 2.3.2 Experimental procedure

The experimental procedure consisted of three phases: an adaptation practice session, a screening test, and the main formal trials. Both the practice and screening test were conducted in a separate scenario distinct from the main trials, lasting 20 and 10 min, respectively. These initial phases aimed to familiarize participants with the simulator controls, ensure their ability to independently perform basic driving tasks (e.g., lane keeping, speed control, acceleration/deceleration, lane changing, and sign recognition), and screen out individuals who experienced significant simulator discomfort.

The formal experiment used a randomized design where each of the four scenarios was presented in a random order. Each participant therefore completed four trial runs, one for each scenario. A 20-min break was enforced between trials to mitigate learning effects. Participants were not informed of the specific research objectives.

The formal trial procedure was as follows: (1) Prior to the first trial, participants completed a questionnaire providing personal information (e.g., gender, age, freeway driving mileage); (2) They were instructed that their destination was “City B” and were told to follow all informational guidance presented in the scenario. (3) They then completed all four experimental trials. A key instruction was that participants must remain in the left-most lane until they received the closure information. This design was based on two considerations. First, on multi-lane freeways in China, the left-most lane typically has the highest speed limit (110–120 km/h) and is commonly used by passenger cars. Second, exiting from the left-most lane requires four lane changes, representing the most behaviorally demanding and complex maneuver, thereby facilitating comprehensive observation of driving behavior characteristics.

Upon completion of the experiment, each participant received compensation of 200 CNY, which exceeded the local average daily wage. Throughout all trials, the driving simulator recorded data on driving operations, vehicle dynamics, driving performance, position, and time at a frequency of 10 Hz. All data were saved in log file format after each session.

### 2.4 Driving behavior evaluation indicator system

To quantitatively assess how warning signs at the entrance of a closure control zone regulate driving behavior under different weather conditions, this study developed a set of indicators based on the driver’s core tasks in this scenario. These indicators are selected to directly reflect the safety-related aspects of both lateral (steering) and longitudinal (speed control) vehicle operations, thereby enabling a systematic evaluation of the effects of sign content and environmental factors.

Within the control zone, drivers must execute a series of mandatory actions: laterally, they need to perform three consecutive lane changes to transition from the leftmost to the rightmost lane; longitudinally, they must smoothly decelerate from the mainline speed to the ramp speed limit following graded speed limit signs. Based on this task analysis, the evaluation framework (Table 2) focuses on two key dimensions of behavioral performance that are critical to safety in this specific scenario:

1. Timeliness and Spatial Redundancy of Maneuvers.
2. Timeliness of Lane Change Initiation (*X_s_*): Measures the distance from the influence zone origin to the point where the driver initiates the first lane change. Earlier initiation indicates that the warning sign effectively prompts drivers to perceive the hazard and begin planning, thereby providing more spatial margin for subsequent maneuvers and reducing last-minute urgent decisions.
3. Redundancy of Lane Change Completion (*X_e_*): Measures the distance to the reference point when the final lane change is completed. Greater redundancy means drivers finish the lane-change sequence earlier, leaving additional buffer space to absorb unexpected events. These two metrics together test whether the warning signs encourage proactive rather than reactive behavior.
4. Dynamic Characteristics of the Maneuver Process.

1. Aggressiveness of Lane Changes (*L_ys1_*, *L_ys2_*, *L_ys3_*): Quantified by the average yaw rate during the 2-s period immediately preceding full entry into the target lane. Aggressive lane changes increase the risk of sideswipe collisions and disrupt traffic flow in adjacent lanes. This metric captures the intensity of lateral motion during the most critical conflict phase, making it a direct indicator of safety risk.
2. Deceleration Aggressiveness (*A_m_*): Defined as the maximum absolute deceleration observed within the control zone. Abrupt braking reflects high instantaneous risk and can lead to rear-end collisions.
3. Deceleration Smoothness (*V_v_*): Represented by the time-varying stochastic volatility of speed throughout the deceleration phase. A smoother speed profile indicates better anticipation and control, reducing the likelihood of abrupt speed changes and improving overall traffic stability.

**Table 2:** Driving behavior evaluation indicator system.

Indicator category	Indicators	Description	Notes
Lateral behavior	Timeliness of lane change initiation (*Xs*) / m	The distance from the Influence Zone Origin of the closure control zone to the point where the vehicle initiates its first lane change.	The Influence Zone Origin is located 250 meters upstream of the closure control zone proper, where drivers first see the warning sign and become influenced by the closure. A smaller *Xs* value indicates that the driver initiated the lane change response earlier.
	Redundancy of lane change completion (*Xe*) / m	Distance to the reference point when the vehicle completes the final lane change.	Larger *Xe* indicates greater maneuvering redundancy.
	Aggressiveness of 1st lane change (*L_ys1_*) / (°/s)	Average yaw rate in the 2 s prior to fully entering the target lane during the first lane change.	A larger absolute value indicates more aggressive steering.
	Aggressiveness of 2nd lane change (*L_ys2_*) / (°/s)	Average yaw rate in the 2 s prior to fully entering the target lane during the second lane change.	
	Aggressiveness of 3rd lane change (*L_ys3_*) / (°/s)	Average yaw rate in the 2 s prior to fully entering the target lane during the third lane change.	
Longitudinal behavior	Deceleration aggressiveness (*A_m_*) / (m/s^2^)	Maximum absolute deceleration value observed within the control zone.	A larger absolute value indicates more intense/abrupt braking.
	Deceleration smoothness (*V_v_*)	Time-varying stochastic volatility of speed throughout the deceleration phase within the control zone.	A larger value indicates greater speed fluctuation and less smooth control.

The combination of these indicators allows a comprehensive assessment of how warning sign content and weather conditions influence both the planning (timeliness, redundancy) and execution (aggressiveness, smoothness) of driving maneuvers, directly aligning with the research objective of mitigating behavioral risks in complex eight-lane freeway closures.

Details regarding the construction of this indicator system are explained below:

1. Behavioral Event Identification.

To accurately identify the start and end of lane-changing and deceleration events, a threshold-based method using motion parameters was employed (26, 27) Equation 1. The onset was defined as the moment the vehicle’s lateral velocity first exceeded 0.15 m/s. The completion was marked when the lateral velocity fell below 0.15 m/s for at least 1 s after entering the adjacent lane. The onset was defined as the point when deceleration became consistently less than −0.1 m/s^2^ for at least 2 s. The end was marked when deceleration subsequently remained greater than −0.1 m/s^2^ for at least 1 s. Lateral velocity was calculated as the differential of the vehicle’s lateral offset from the road centerline.


$$V(t_{i})=\frac{y_{i+1}-y_{i}}{t_{i+1}-t_{i}}$$ (1)

In the formula: The term yᵢ denotes the lateral offset of the vehicle from the road centerline at the i-th time step, and tᵢ represents the corresponding timestamp.

1. Quantification of Lane-Change Aggressiveness.

Given that multiple mandatory lane changes are required in the control zone, aggressive maneuvers can severely disrupt adjacent traffic flow and pose a high risk for sideswipe collisions. Therefore, the aggressiveness of each of the three lane changes (*L_ys1_, L_ys2_, L_ys3_*) was selected as a key metric. This is quantified by the average yaw rate (*L_ys_*) over the 2-s period immediately preceding the vehicle’s full entry into the target lane.

The calculation is as Equation 2:


$$L_{\mathrm{ys}}=\frac{1}{N}\sum_{t=t_{s}}^{t_{e}}\omega (t)\;$$ (2)

Where: ω(t) is the instantaneous yaw rate at time step k (°/s); $t_{s}$ and $t_{e}$ are the start and end times, respectively, of the 2-s period immediately preceding the vehicle’s full entry into the target lane; N is the number of sampling points within this interval. The calculation is based on the yaw rate’s direct representation of lateral motion intensity. This specific 2-s window is considered the critical conflict phase where the merging vehicle poses the highest risk to traffic in the target lane—before this period, the intrusion is minimal, and after it, the merge is largely complete.

1. Quantification of Deceleration Aggressiveness and Smoothness.

Longitudinal safety was evaluated from two complementary perspectives: instantaneous risk and process stability.

1. Deceleration Aggressiveness (*A_m_*): Defined as the maximum absolute deceleration value observed within the control zone, capturing extreme braking intensity.
2. Deceleration Smoothness (*V_v_*): Represented by the Time-varying Stochastic Volatility of Velocity. This metric quantifies the continuity and stability of speed control throughout the deceleration phase by measuring the volatility of speed over time. Lower values indicate a smoother, less fluctuating deceleration profile.

*V_v_* is calculated as Equations 3, 4:


$$r_{i}=\ln(\frac{v_{i}}{v_{i-1}})*100$$ (3)

$$V_{v}=\sqrt{\frac{1}{N-1}\sum {\left(r_{i}-\bar{r}\right)}^{2}}$$ (4)

Where: $v_{i}$ is the speed at time step i-th, N is the number of sampling points; The calculation covers the entire closure control zone.

## 3 Results

To examine the overall differences among the four experimental scenarios (2 sign types × 2 weather conditions), a one-way Analysis of Variance (ANOVA) was conducted for each dependent variable, with experimental scenario as the independent variable (four levels: Warning-Clear, Warning & Advice-Clear, Warning-Dust, Warning & Advice-Dust). This approach allows for direct comparison of driving performance across all condition combinations and is followed by post-hoc pairwise comparisons to identify specific differences between scenarios. Where significant F-tests were found, post-hoc comparisons were conducted using the Least Significant Difference (LSD) method. The results of these pairwise comparisons are indicated by markers in the subsequent figures, where ‘ns’ denotes *p* > 0.05, “denotes *p* ≤ 0.05, “denotes *p* ≤ 0.01, “denotes *p* ≤ 0.001, and ‘****’ denotes *p* ≤ 0.0001. The assumptions of normality and homogeneity of variance were assessed using Kolmogorov–Smirnov test and Levene’s test, respectively, and were satisfied for all indicators. The descriptive statistics and one-way ANOVA results for all indicators are summarized in Table 3. The analysis revealed that all indicators reached statistical significance except for the Redundancy of Lane Change Completion (*X_e_*).

**Table 3:** Descriptive statistics and one-way ANOVA results for driving behavior indicators.

Indicator category	Indicators		Warning—clear	Warning and advice—clear	Warning—dusty	Warning and advice—dusty	F	*p*
Lateral behavior	Timeliness of lane change initiation (*Xs*) / m	Mean	1191.73	225.61	825.34	215.39	47.01	0.000*
		SD	614.61	57.55	444.61	82.33		
		Max	1971.19	353.16	1714.48	414.22		
		Min	275.00	117.36	151.89	81.30		
	Redundancy of lane change completion (*Xe*) / m	Mean	428.46	516.57	554.62	572.57	2.295	0.081
		SD	305.82	188.69	246.54	158.53		
		Max	1033.30	853.86	958.62	924.93		
		Min	47.90	172.74	158.42	247.73		
	Aggressiveness of 1st lane change (*L_ys1_*) / (°/s)	Mean	−1.20	−0.77	−0.73	−0.37	26.86	0.000*
		SD	0.51	0.39	0.29	0.15		
		Max	−0.09	−0.04	−0.08	−0.01		
		Min	−2.38	−1.81	−1.28	−0.72		
	Aggressiveness of 2nd lane change (*L_ys2_*) / (°/s)	Mean	−1.64	−1.25	−1.12	−0.77	8.65	0.000*
		SD	0.96	0.56	0.62	0.43		
		Max	−0.06	−0.29	−0.08	0.00		
		Min	−3.75	−2.65	−2.51	−1.59		
	Aggressiveness of 3rd lane change (*L_ys3_*) / (°/s)	Mean	−2.51	−1.92	−1.88	−1.53	6.20	0.001*
		SD	1.13	0.78	0.92	0.70		
		Max	−0.33	−0.15	0.00	−0.15		
		Min	−4.54	−3.63	−3.54	−3.25		
Longitudinal behavior	Deceleration aggressiveness (*A_m_*) / (m/s^2^)	Mean	−2.03	−1.59	−1.75	−1.31	12.57	0.000*
		SD	0.69	0.50	0.33	0.17		
		Max	3.54	2.51	2.40	1.61		
		Min	0.89	0.85	0.95	0.99		
	Deceleration smoothness (*V_v_*)	Mean	2.38	2.00	1.97	1.47	84.27	0.000*
		SD	0.20	0.25	0.24	0.20		
		Max	2.75	2.42	2.34	1.76		
		Min	1.93	1.59	1.43	1.08		

* Indicates a significant difference.

### 3.1 Lane-changing behavior

#### 3.1.1 Timeliness of lane change initiation

**Figure 5:**
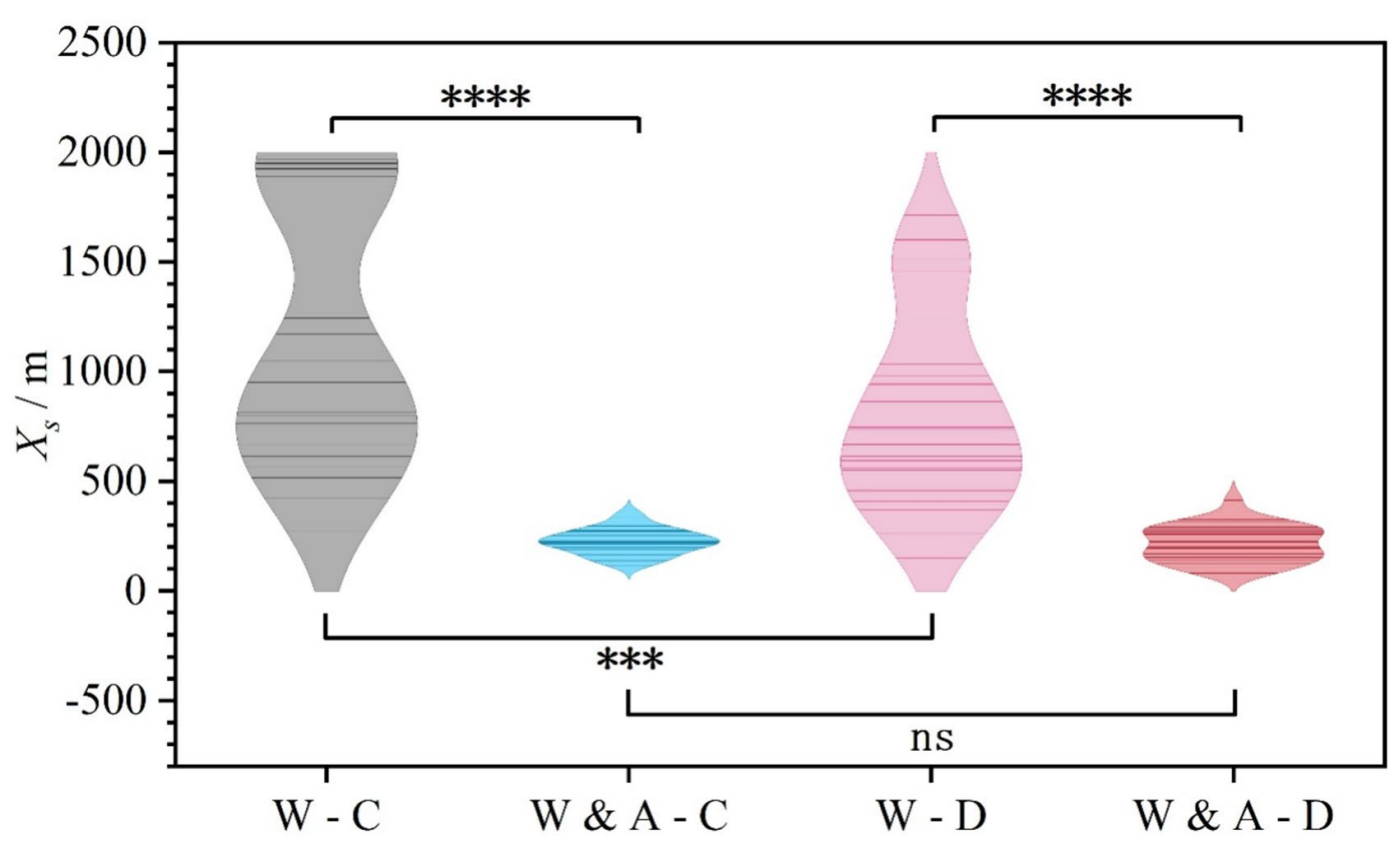
Comparison of lane change initiation timeliness (*X*_s_) across experimental scenarios.

As shown in Figure 5 and Table 3, the results for the Timeliness of Lane Change Initiation (*X_s_*) clearly reveal a significant influence of both warning sign content and weather condition on drivers’ initial response behavior (*F* = 47.01, *p* < 0.001).

The key patterns are as follows. First, information content was the primary factor determining response timeliness. The mean *X_s_* values for the “Warning & Advice” sign group (Clear: 225.61 m; Dust: 215.39 m) were substantially smaller than those for the basic “Warning” sign group (Clear: 1191.73 m; Dust: 825.34 m) under both weather conditions. This indicates that signs containing the specific advisory “Keep Right to Exit” effectively prompted drivers to begin planning and executing lane changes almost immediately upon seeing the sign. Second, informational clarity ensured consistent responses. The standard deviation of *X_s_* was significantly larger for the basic “Warning” group (Clear: 614.61 m; Dust: 444.61 m) than for the “Warning & Advice” group (Clear: 57.55 m; Dust: 82.33 m). This stark contrast suggests that ambiguous information led to highly divergent response strategies among drivers: some responded promptly at the beginning of the influence zone, while others delayed until compelled by downstream channelizing devices. Furthermore, adverse weather itself had an alerting effect. Comparing weather conditions within the same sign type shows that dusty weather prompted drivers to initiate lane changes earlier. For instance, with only the basic “Warning” sign, the mean response distance in dusty weather (825.34 m) was approximately 366 meters earlier than in clear weather (1191.73 m). In summary, providing a warning sign with explicit action instructions at the control zone entrance is an effective strategy for ensuring timely and consistent initiation of lane changes.

#### 3.1.2 Redundancy of lane change completion

**Figure 6:**
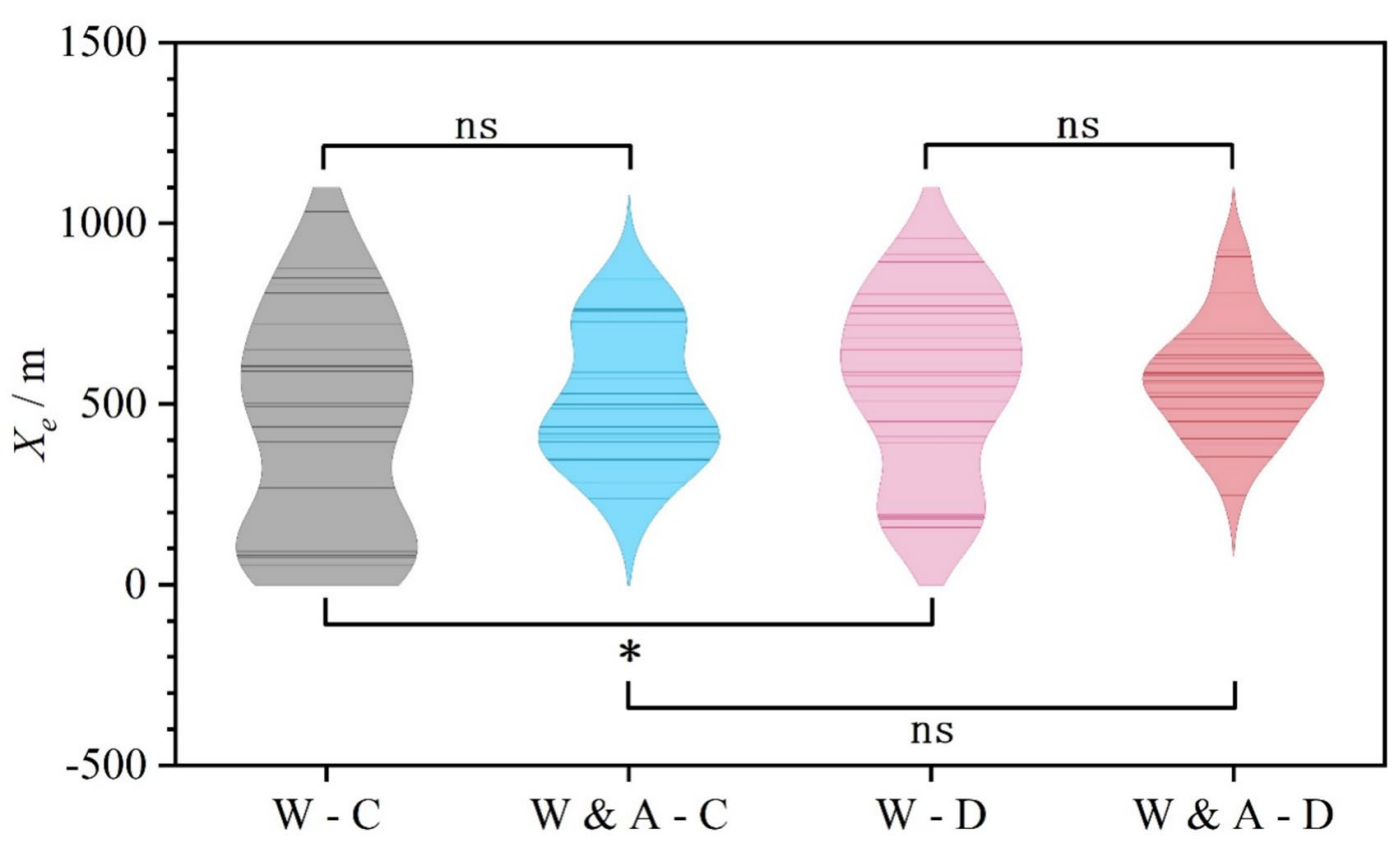
Comparison of lane change completion redundancy (X_e_) across experimental scenarios.

Statistical analysis of the Redundancy of Lane Change Completion (*X_e_*) showed that the differences between experimental scenarios did not reach statistical significance (*F* = 2.295, *p* = 0.081), as shown in Figure 6 and Table 3.

However, a closer examination of the descriptive statistics reveals an important behavioral pattern: information content influenced the concentration and consistency of lane change completion. Specifically, in scenarios with only the basic “Warning” sign (especially “Warning-Clear”), *X_e_* had the smallest mean (428.46 m) but the largest standard deviation (305.82 m), with the data distribution suggesting a bimodal trend. This indicates that in the absence of clear guidance, driver behavior was highly polarized. Some drivers completed the lane change far upstream from the base point, while others did so only when approaching it, resulting in low overall redundancy with high instability. In contrast, under the guidance of the “Warning & Advice” sign, the mean *X_e_* increased across scenarios and the standard deviation decreased significantly. This shows that while the explicit advisory did not statistically alter the average completion point, it effectively narrowed the behavioral distribution, leading more drivers to adopt a more consistent and moderately earlier lane-changing strategy, thereby creating overall better and more predictable operational redundancy.

#### 3.1.3 Aggressiveness of lane-change maneuvers

**Figure 7:**
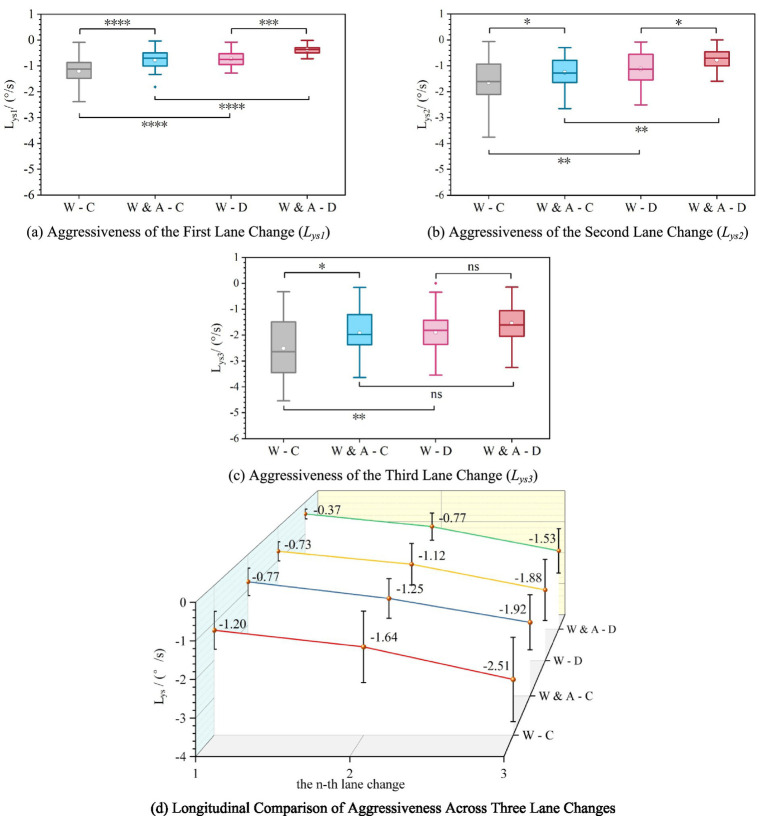
Aggressiveness of lane-change maneuvers. Subfigures **(a–c)** are bar charts comparing the aggressiveness of the first, second, and third lane change, respectively, across scenarios. Subfigure **(d)** is a line chart plotting the mean aggressiveness for all three lane changes sequentially, showing that aggressiveness increases with each successive change and is consistently lower for the “Warning & Advice” groups. All charts include error bars and significance markers.

Statistical analysis of the aggressiveness of the three lane-change maneuvers indicates that both warning sign content and the sequence of the lane change significantly influenced driving behavior. As shown in Figures 7a–d and Table 3, the overall pattern can be summarized as follows: the “Warning & Advice” sign effectively reduced the aggressiveness of each lane change, with this effect being more pronounced in dusty weather. Furthermore, maneuver aggressiveness increased significantly with each successive lane change, but the “Warning & Advice” sign effectively mitigated this increasing trend.

Specifically, under the same weather condition, the “Warning & Advice” sign reduced the aggressiveness of the three lane changes by 35.8, 23.8, and 23.5% in clear weather, and by 49.3, 31.3, and 18.6% in dusty weather, compared to the basic “Warning” sign. This demonstrates that explicit behavioral guidance effectively promotes smoother lane changes, although its guiding effect diminishes with successive maneuvers. The between-group difference for the third lane change in dusty weather was not significant, reflecting that the increased operational urgency due to spatial constraints in the later stage of the task weakened the independent influence of information type. From a sequential perspective, the “Warning & Advice” sign significantly flattened the growth in aggressiveness across the task. For example, in clear weather, the total increase in aggressiveness from the first to the third lane change was smaller in the “Warning & Advice” group (1.15 °/s) than in the basic “Warning” group (1.31 °/s). This proves that the sign, through its proactive guidance, optimized drivers’ planning of the entire lane-change sequence, shifting their approach from “reactive” to “proactive,” thereby systematically enhancing the smoothness and controllability of serial lane-changing behavior.

### 3.2 Deceleration behavior

#### 3.2.1 Deceleration aggressiveness

**Figure 8:**
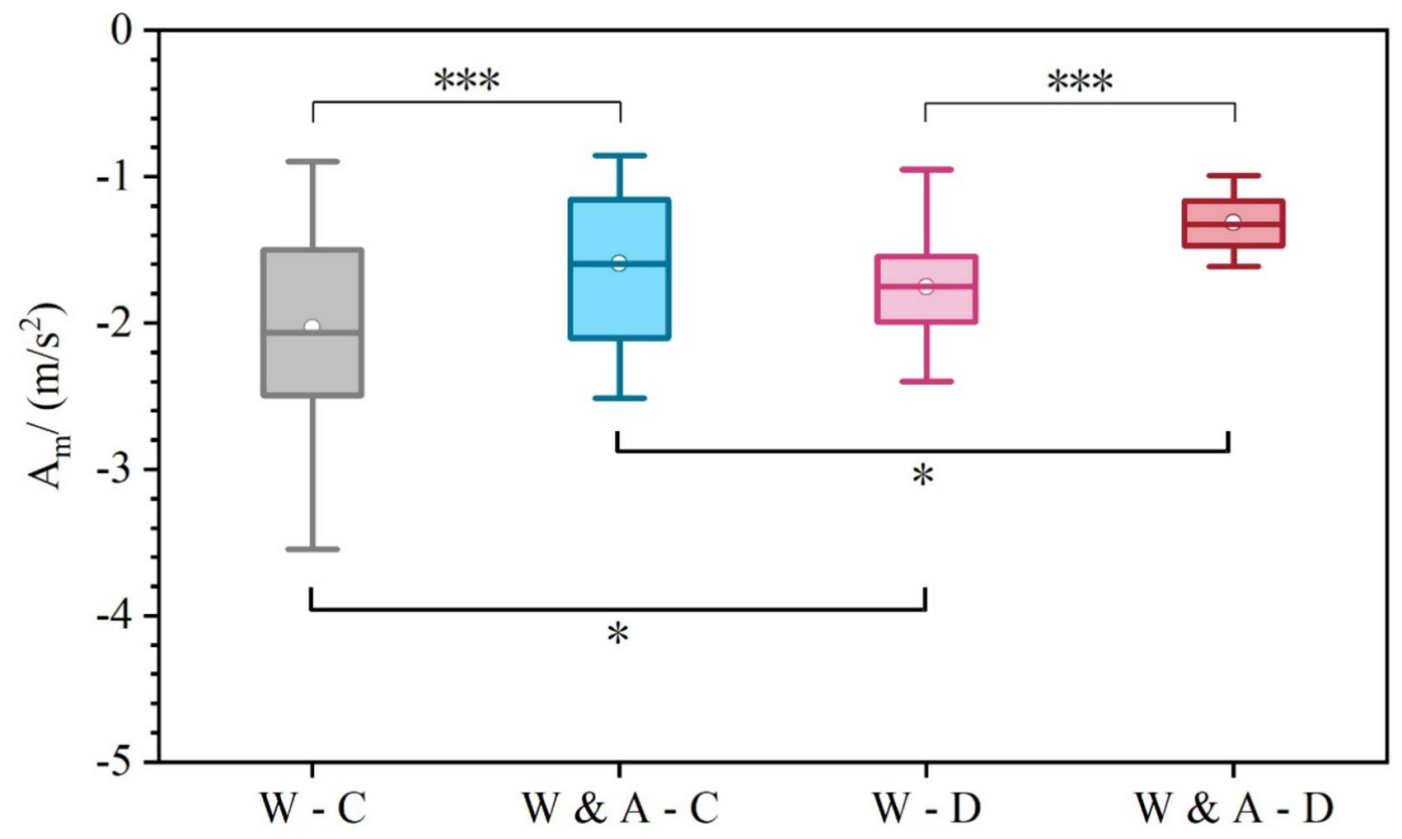
Comparison of deceleration aggressiveness (maximum absolute deceleration A_m_ in m/s^2^) across experimental scenarios.

As shown in Figure 8 and Table 3, the results for Deceleration Aggressiveness (*A_m_*) reveal a significant influence of both warning sign content and weather condition on drivers’ braking intensity (*F* = 12.57, *p* < 0.001). The mean absolute values of this metric across scenarios, ranked from highest to lowest, are: Warning-Clear (2.03 m/s^2^) > Warning-Dust (1.75 m/s^2^) > Warning & Advice-Clear (1.59 m/s^2^) > Warning & Advice-Dust (1.31 m/s^2^). The results indicate that the “Warning & Advice” sign effectively mitigated the aggressiveness of deceleration behavior under both weather conditions, and that braking maneuvers were gentler in dusty weather.

Regarding the influence of different sign contents under the same weather condition: In clear weather, the absolute Am value for the “Warning & Advice” group (1.59 m/s^2^) was 21.7% lower than that for the “Warning” group (2.03 m/s^2^), indicating that the advisory information effectively guided drivers to decelerate more smoothly. This advantage was even greater in dusty weather, where the “Warning & Advice” group (1.31 m/s^2^) showed a 25.1% reduction compared to the “Warning” group (1.75 m/s^2^). This highlights the increased importance of proactive, instructional information for improving deceleration safety under low-visibility conditions. Regarding the influence of different weather conditions under the same sign content: When only the “Warning” sign was used, the Am value in dusty weather (1.75 m/s^2^) was 13.8% lower than in clear weather (2.03 m/s^2^), confirming that adverse weather itself prompts more cautious longitudinal control. When the “Warning & Advice” sign was used, the value in dusty conditions (1.31 m/s^2^) showed a further 17.6% decrease compared to clear conditions (1.59 m/s^2^). This suggests that the alerting effect of weather and the guiding effect of the advisory information worked synergistically to produce the gentlest deceleration behavior.

#### 3.2.2 Deceleration smoothness

**Figure 9:**
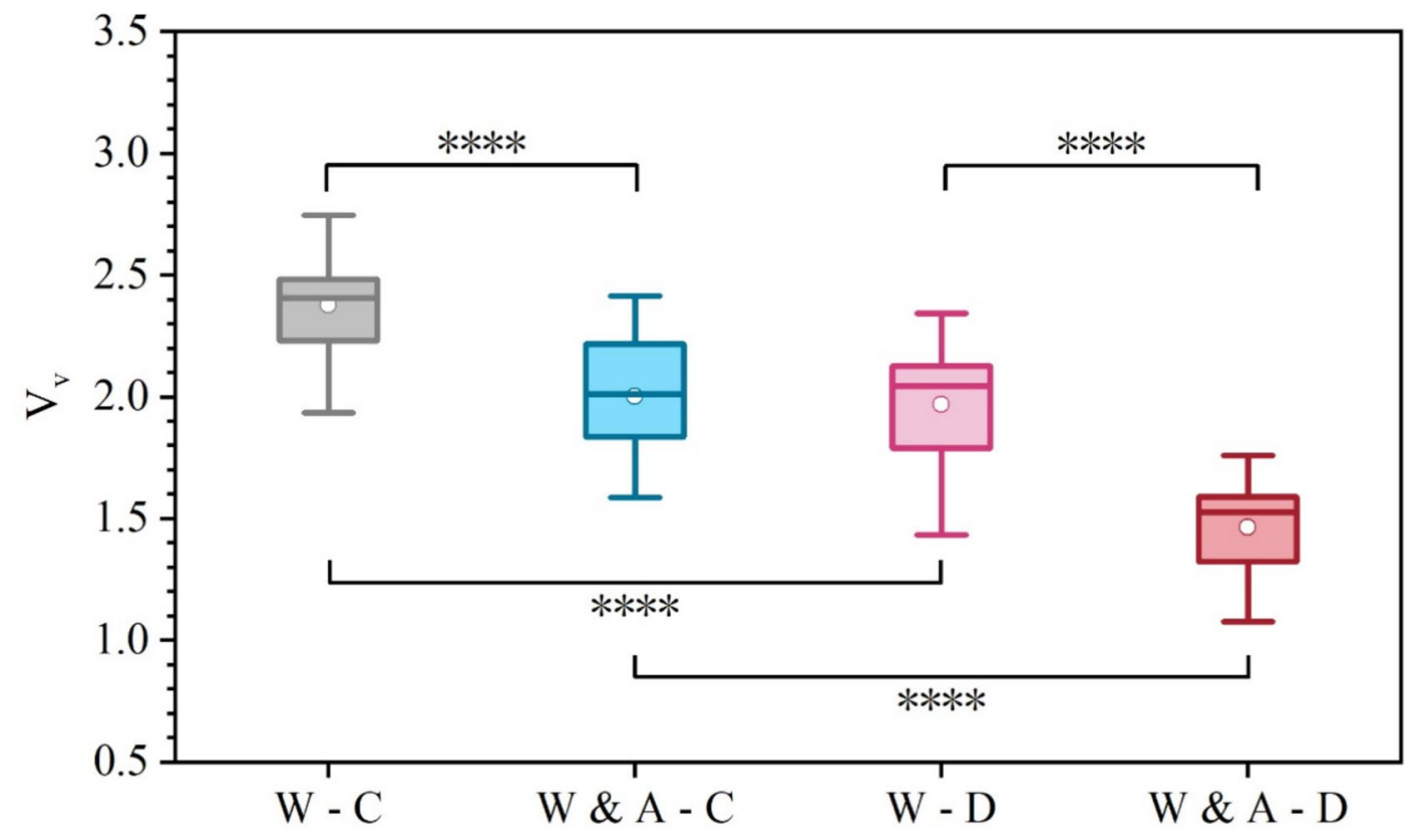
Comparison of deceleration smoothness (volatility index Vv) across experimental scenarios.

As shown in Figure 9 and Table 3, the results for Deceleration Smoothness (*V_v_*) reveal a significant influence of both warning sign content and weather condition on the stability of speed control (*F* = 84.27, *p* < 0.001). The mean values of this metric across scenarios, ranked from highest to lowest, are: Warning-Clear (2.38) > Warning & Advice-Clear (2.00) > Warning-Dust (1.97) > Warning & Advice-Dust (1.47). This order indicates that the “Warning & Advice” sign effectively improved the smoothness of the deceleration process, and that the process was smoother in dusty weather.

Regarding the influence of different sign contents under the same weather condition: In clear weather, the *V_v_* value for the “Warning & Advice” group (2.00) was significantly lower than that for the “Warning” group (2.38), a reduction of 16.0%. This shows that adding advisory information helped drivers develop a more stable and coherent speed control strategy, reducing unnecessary speed adjustments. This advantage expanded in dusty weather, where the “Warning & Advice” group (1.47) showed a 25.4% reduction compared to the “Warning” group (1.97), underscoring drivers’ heightened reliance on guidance information in adverse weather and demonstrating that optimized information content can significantly suppress speed fluctuations, enhancing longitudinal smoothness and safety. Regarding the influence of different weather conditions under the same sign content: When the “Warning” sign was used, the *V_v_* value in dusty weather (1.97) was 17.2% lower than in clear weather (2.38), further verifying the promoting effect of adverse weather itself on smoother driving. When the “Warning & Advice” sign was used, the *V_v_* value in dusty weather (1.47) decreased by 26.5% compared to clear conditions (2.00), an effect greater than the 17.2% observed with the basic sign. This indicates a synergistic interaction between the alerting effect of weather and the guiding effect of information, jointly facilitating the smoothest deceleration process.

## 4 Discussion

Addressing the lack of dedicated standards for mainline closure control on eight-lane freeways during emergencies, this study proposed a systematic “Closure Control Zone” design concept. Through a 2 × 2 factorial driving simulation experiment, it specifically investigated the effects of warning sign content (Warning vs. Warning & Advice) and weather conditions (Clear vs. Dust) on driving behavior safety. The core finding demonstrates that providing a warning sign with action advice at the zone entrance consistently and significantly reduces the aggressiveness of both lane-changing and deceleration maneuvers, an effect particularly pronounced under adverse dusty weather conditions. This discussion first examines the alignment of our results with existing literature, then analyzes unexpected findings and their theoretical implications, and finally clarifies the study’s limitations.

The present findings align with and extend several conclusions from prior research on traffic sign effectiveness. First, this study confirms the superiority of providing specific action advice, which is highly consistent with the findings of Bham et al. (4), who reported that strong semantic messages like “Prepare to Stop” elicited the most substantial speed reductions. The high magnitude of improvement observed with the “Warning & Advice” sign in our study (e.g., a 49.3% reduction in first lane-change aggressiveness under dust) further indicates that in the more complex decision-making context of a full mainline closure, an explicit instruction (“Keep Right to Exit”) provides drivers with a clear behavioral script, effectively reducing decision hesitation and operational delays (4).

Second, the mitigating effect of adverse weather on driving behavior observed here corroborates reports by Yang et al. (7) and Hang et al. (8), namely that drivers spontaneously adopt more cautious strategies under low-visibility conditions. An important advancement of this study, however, lies in revealing the interaction between weather and information type: dusty weather not only amplified the benefits of the “Warning & Advice” sign but also prompted safer behavior even when only the basic warning was provided. This supports the “Risk Compensation” theory in human factors, whereby increased perceived external risk prompts individuals to adjust their behavior to maintain a subjective feeling of safety.

Nevertheless, one detail appears partially inconsistent with some existing literature. The between-group difference for the “Warning & Advice” sign became non-significant during the third lane change in dusty weather. This seems to contradict the general conclusion from studies like Duan et al. (14) and Hang et al. (13) that “earlier prompts yield better effects.” We posit three potential explanations: (1) As the lane-change sequence progresses, cumulative task load may saturate drivers’ attentional resources, diminishing their capacity to process additional advisory information. (2) During later lane changes, vehicles are already positioned further rightward, and increased spatial constraints may limit maneuvering flexibility, compelling more urgent lateral movement regardless of sign content. (3) The influence of the sign, placed only at the zone entrance, may naturally attenuate over time and distance, suggesting a potential need for repeated or reinforced messages within the control zone in future designs.

A key serendipitous finding was that when the “Warning & Advice” sign was used, the behavioral differences between dusty and clear weather conditions became non-significant. This contradicts the intuitive assumption that adverse weather invariably alters behavior significantly. A plausible explanation is that high-quality informational guidance may partially “compensate” for the uncertainty introduced by a hostile environment. When a sign provides a sufficiently clear and credible action plan, it reduces the driver’s need to self-explore safe strategies in poor weather, causing their behavior to approximate the guided state observed in ideal conditions. This implies that optimizing information design could be an effective strategy for mitigating the negative impacts of adverse weather on driving.

The results necessitate an important revision and extension of existing work zone control models. Traditional standards (e.g., JTG-H30, MUTCD) are primarily based on principles of physical separation and static warning, with information design often focusing on “risk notification” rather than “action guidance.” Our findings strongly suggest that for high-complexity scenarios like freeway closures, the control paradigm should evolve from a “Risk-Warning” model to a “Task-Guidance” model.

Based on our results, we propose a core concept for an information-behavior interaction model within closure control zones: the safety benefit stems not only from the physical enforcement of channelization but, crucially, from how upfront information optimizes the driver’s cognitive-decision process. An effective warning sign, by delivering a complete information chain—specifying what (the hazard), why (the closure), and how (the advised action)—can proactively intervene during the task-planning stage. This shifts the driver’s operational mode from reactive response to proactive planning. This cognitive shift serves as the key psychological mechanism underlying the observed smoother lane changes and more linear deceleration profiles. The model frames driving safety as the dynamic interaction of environmental conditions, engineering design, and information comprehension, thereby providing a theoretical foundation for the future refined and intelligent design of closure control zones.

From a practical perspective, the findings offer several actionable recommendations for real-world implementation. First, traffic management agencies should consider incorporating explicit action advice—such as “Keep Right to Exit” or “Merge Now”—into warning signs for emergency mainline closures, particularly on multi-lane freeways where multiple lane changes are required. The enhanced sign tested in this study provides a template that can be adapted to local contexts and emergency types (e.g., “Fog Ahead—Exit Right” or “Accident Closure—Prepare to Merge”). Second, the observed attenuation of sign effects during later lane changes suggests that a single upstream warning may be insufficient; supplemental reinforcement—such as repeating the advisory message on downstream variable message signs or incorporating pavement markings—could help sustain driver compliance throughout the control zone. Third, the amplified benefits under dusty weather highlight the importance of dynamic information strategies that adapt to environmental conditions. Agencies could integrate weather monitoring systems with variable message signs to trigger enhanced warnings when visibility drops below critical thresholds. Finally, the proposed control zone design and sign content can inform updates to national standards (e.g., GB-5768, JTG-H30, MUTCD) by providing empirical evidence for dedicated full-closure protocols on high-grade highways.

This study inevitably has several limitations. First, while the sample size of 30 participants aligns with common practice in driving simulation studies (1, 2) and meets basic statistical power requirements, a larger sample would enhance the robustness of the conclusions and permit finer-grained analysis of driver subgroups (e.g., by driving experience, gender). Second, although the driving simulator provides a high-fidelity and controlled environment, differences in risk perception and physiological load compared to real-world driving may mean the observed behavioral mitigation effects are somewhat attenuated in actual scenarios (28). Future research could extend these aspects to comprehensively evaluate the generalizability of the proposed closure control zone scheme and warning signs.

## 5 Conclusion

This study addresses the practical challenge of lacking dedicated control schemes for emergency mainline closures on eight-lane freeways. It introduces the design concept and methodology of a “Closure Control Zone,” with a specific focus on the information content of warning signs at the zone entrance. Through a high-fidelity driving simulation experiment, the mechanisms by which different information levels and weather conditions influence driving safety were elucidated. The principal findings are as follows:

1. Compared to a traditional warning sign, incorporating the “Keep Right to Exit” advisory significantly improved the timeliness, redundancy, and smoothness of driving maneuvers. Specifically, in clear weather, this sign led drivers to initiate lane changes approximately 966 meters earlier, reduced the aggressiveness of three successive lane changes by 35.8, 23.8, and 23.5% respectively, decreased deceleration aggressiveness by 21.7%, and improved deceleration smoothness by 16.0%. Under dusty conditions, it prompted lane change initiation 610 meters earlier, reduced the aggressiveness of the first two lane changes by 49.3 and 31.3%, lowered deceleration aggressiveness by 25.1%, and enhanced deceleration smoothness by 25.4%.
2. Clear guidance enhances behavioral consistency. In the absence of specific action advice, drivers’ operational strategies diverged significantly, evidenced by increased dispersion in both lane-change initiation points and completion positions. The “Warning & Advice” sign, by providing explicit behavioral guidance, effectively unified drivers’ operational patterns and improved behavioral predictability.
3. The beneficial effect of adding action advice to warning signs is more pronounced under adverse weather. The “Warning & Advice” sign exerted its most substantial moderating effect under these conditions, indicating that optimized information design is a key strategy for enhancing the environmental robustness of emergency control measures.

The findings offer practical guidance for implementation. First, traffic agencies can adopt the proposed control zone design and enhanced “Warning & Advice” sign—combining hazard identification, closure information, and explicit action guidance—as a template adaptable to various emergency scenarios. Second, the results support integrating weather-responsive strategies, enabling dynamic warning activation when visibility deteriorates. Third, the empirical evidence provides quantitative support for revising national and international standards to incorporate active guidance principles for complex multi-lane closures. This work advocates a shift in control philosophy from “risk notification” to “task guidance,” aiming to optimize driver decision-making and thereby improve the operational effectiveness of high-grade highways under extreme conditions.

## Data Availability

The datasets presented in this article are not readily available because the original data generated for this study are considered confidential and the authors are not authorized to share them publicly. Requests to access the datasets should be directed to 1053756951@qq.com.
